# Supplementary material for: Identification of photoperiod-induced specific miRNAs in the adrenal glands of Sunite sheep (Ovis aries)
Source: Front Vet Sci. 2022 Jul 22;9:888207. doi: 10.3389/fvets.2022.888207 (PMC9354845; doi:10.3389/fvets.2022.888207)
Supplement: Supplementary file 1 [file Data_Sheet_1.ZIP › Supplementary file/miRDeep2 file.zip!/miRDeep2 file/Novel_Structure/SPLP42A2/Novel_658.pdf]

Provisional ID	:	NC_019484.2_43466
Score total	:	1.9
Score for star read(s)	:	-1.3
Score for read counts	:	0
Score for mfe	:	1.6
Score for randfold	:	1.6
Score for cons. seed	:	
Total read count	:	1088
Mature read count	:	1088
Loop read count	:	0
Star read count	:	0

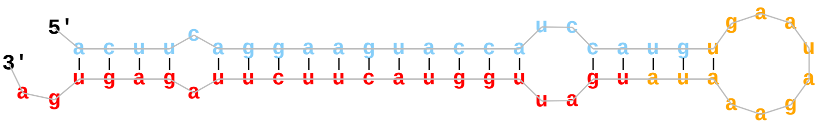

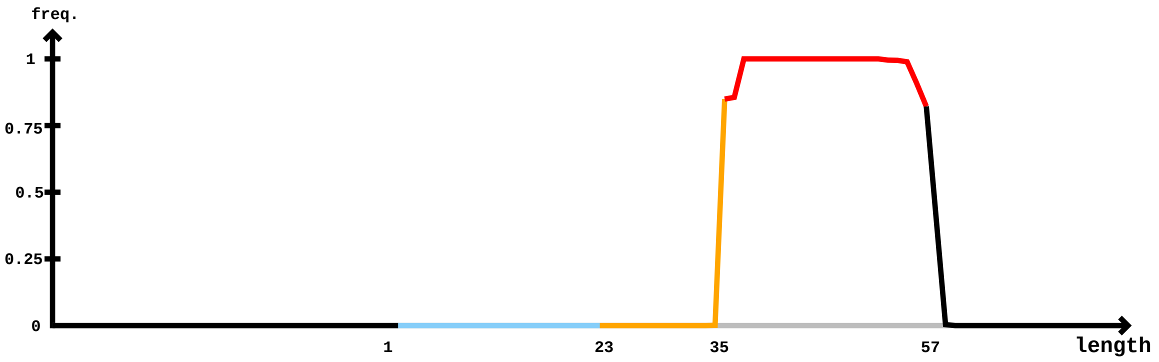


```
                                                                    Star                                        Mature
5'- ucaccacauuucacuaugccgcgugcaguauugcacuucaggaaguaccauccaugugaauagaaauaugauugguacuucuuagagugaaguaaugcauagcaaauaca   -3'    exp
    ................(((...(((((.((((.(((((.(((((((((((..(((((........)))))..))))))))))).))))).)))).))))).)))......    reads    mm    sample
    ...................................................................augauugguacuucuuagagu......................    1        0     seq
    ....................................................................ugauugguacuucuuag.........................    5        0     seq
    ....................................................................ugauugguacuucuuaga........................    1        0     seq
    ....................................................................ugauugguacuucuuagag.......................    5        0     seq
    ....................................................................ugauugguacuucuuagagu......................    85       0     seq
    ....................................................................ugauugguacuucuuagagug.....................    91       0     seq
    ....................................................................ugauugguacuucuuagaguga....................    677      0     seq
    ....................................................................ugauugguacuucuuagagugaa...................    58       0     seq
    ....................................................................ugauugguacuucuuagagugaag..................    1        0     seq
    .....................................................................gauugguacuucuuagagu......................    1        0     seq
    .....................................................................gauugguacuucuuagaguga....................    5        0     seq
    .....................................................................gauugguacuucuuagagugaa...................    1        0     seq
    ......................................................................auugguacuucuuagag.......................    1        0     seq
    ......................................................................auugguacuucuuagagu......................    1        0     seq
    ......................................................................auugguacuucuuagagug.....................    3        0     seq
    ......................................................................auugguacuucuuagaguga....................    110      0     seq
    ......................................................................auugguacuucuuagagugaa...................    39       0     seq
    ......................................................................auugguacuucuuagagugaag..................    3        0     seq
```
